# Methyl Methacrylate (MMA) Treatment of Empty Fruit Bunch (EFB) to Improve the Properties of Regenerated Cellulose Biocomposite Films

**DOI:** 10.3390/polym12112618

**Published:** 2020-11-06

**Authors:** Salmah Husseinsyah, Nur Liyana Izyan Zailuddin, Azlin Fazlina Osman, Chew Li Li, Awad A. Alrashdi, Abdulkader Alakrach

**Affiliations:** 1 Faculty of Chemical Engineering Technology, Universiti Malaysia Perlis (UniMAP), Arau 02600, Perlis, Malaysia; irsalmah@unimap.edu.my (S.H.); liyana.babyblue@yahoo.com (N.L.I.Z.); lilichew1012@gmail.com (C.L.L.); 2 Biomedical and Nanotechnology Research Group, Center of Excellence Geopolymer and Green Technology (CEGeoGTech), Universiti Malaysia Perlis (UniMAP), Arau 02600, Perlis, Malaysia; 3 Chemistry Department, Umm Al-Qura University, Al-qunfudah University College, Al-qunfudah Center for Scientific Research (QCSR), Al Qunfudah 21962, Saudi Arabia; aarashdi@uqu.edu.sa; 4 Chemistry Department, Faculty of Pharmacy, Qasyoun Private University, Damascus 20872, Syria; abdoakrash@hotmail.com

**Keywords:** empty fruit bunch, regenerated cellulose, ionic liquid, methyl methacrylate

## Abstract

The empty fruit bunch (EFB) regenerated cellulose (RC) biocomposite films for packaging application were prepared using ionic liquid. The effects of EFB content and methyl methacrylate (MMA) treatment of the EFB on the mechanical and thermal properties of the RC biocomposite were studied. The tensile strength and modulus of elasticity of the MMA treated RC biocomposite film achieved a maximum value when 2 wt% EFB was used for the regeneration process. The treated EFB RC biocomposite films also possess higher crystallinity index. The morphology analysis indicated that the RC biocomposite film containing MMA treated EFB exhibits a smoother and more homogeneous surface compared to the one containing the untreated EFB. The substitution of the –OH group of the EFB cellulose with the ester group of the MMA resulted in greater dissolution of the EFB in the ionic liquid solvent, thus improving the interphase bonding between the filler and matrix phase of the EF RC biocomposite. Due to this factor, thermal stability of the EFB RC biocomposite also successfully improved.

## 1. Introduction

Biopolymers that derive from renewable resources have sparked great interest to the world community due to their benefits to the environment and sustainability. Their properties and characteristics demonstrated capability as substitutes for the common traditional petrochemical plastics for various applications [1,2,3,4]. Biopolymers are plenty and can be derived from various sources. Generally, biopolymers can be divided into poly (amino acids) and proteins, poly-, di- and monosaccharides such as chitin [5], starch, glucose, fructose and cellulose [6,7]. Cellulose which have the formula of (C_6_H_10_O_5_)_n_ are recognized as renewable materials that can be found naturally abundant on Earth. These natural resources exist vastly in plants, animals and some microorganisms and can cover a range or variety of properties for instance from biodegradable and biocompatibility to being economical and low cost [5,6,7,8]. They are now considered as one of the most promising polymeric materials with their derived products applicable to numerous parts and sectors, principally in fibers, polymers, paints, papers and films industry [5,6]. Despite such outstanding characteristics shown, there are also some limitations or disadvantages that are raised specifically in terms of cellulose processing. In order to produce cellulosic based film, the cellulose needs to be dissolved prior to the casting process. However, the dissolution process of cellulose is challenging since cellulose are known to be non-soluble in common solvents (water and conventional organic solvents). The insolubility of cellulose is due to the presence or appearance of intra- and intermolecular hydrogen bonding and Van der Waals interaction which closely pack the chains together, and also the partially crystalline structure of cellulose [8,9]. These restraints have motivated further discoveries and investigations for suitable cellulose dissolution methods to facilitate or aid in the use of cellulose, for example, in applications of regenerated cellulose [8,10,11]. Regenerated cellulose (RC) can be referred to as the chemical dissolution of insoluble natural cellulose followed by the recovery of the material from the solution [10,11]. Regenerated celluloses (RCs) are produced or formed commercially using various methods, for example, cellulose carbamate process, lyocell process and viscose process. These different routes can result in fibers with a range of mechanical properties like fiber with low elongation but high tenacity and modulus, and fiber with great elongation but low or small modulus and tenacity [10,11]. Ionic liquids (ILs) are recognized as purely ionic, salt-like materials that are liquid at unusually low temperatures or in simple context they are ionic compounds which are liquid below 100 °C [12,13]. ILs demonstrate exceptional performance or behavior for a variety of applications in the chemical industry, for instance catalysts, chemical synthesis, separation and preparation of materials [14]. Xia et el. have recently reviewed recent advances in processing and volarization of lignocellulosic materials in ILs, including the use of ILs as pre-treatment solvents for lignocellulose to improve and enhance accessibility for lignocellulose-based biocomposite production. They summarized that current research trends have shown that the ILs have a huge potential in multifarious, efficient and environmentally friendly utilization of lignin, lignocellulose and cellulose in green technology advancement. By utilizing ILs as the solvent, additive and dispersant, the production of high performance materials generated from natural plants or biomass materials can be increased and applied in various industries and fields [15]. Other findings also agreed that ILs are considered as an adequate solvent to dissolve cellulose for the cellulose regeneration process and this approach be employed for advanced applications [10,12]. The recovery of ILs after cellulose regeneration could be achieved via methods like evaporation, reverse osmosis, ion exchange and salting out for reuse and recycling purposes [16]. Other solvents that can be used for dissolving cellulose to generate regenerated cellulose are N-methylmorpholine-N-oxide (NMMO) [17], N-dimethylacetamide (DMAC) / lithium chloride (LiCl) [10], 1-butyl-3-methylimidazolium chloride (BMIMCl) [18], 1-allyl-3-methylimidazolium chloride (AMIMCl) [19], 1-ethyl-3-methylimidazolium acetate (EMIMAc) [20] and 1-ethyl-3-methylimidazolium chloride (EMIMCl) [21]. The solvent 1-butyl-3-methylimidazolium chloride (BMIMCl) was first determined or established by Swatloski and co-workers to have good dissolving power for cellulose [22]. This finding generates a way towards a new class of cellulose solvent system, and accelerates or prompts the investigation into the potential of other ILs in dissolving cellulose. 

Oil palm empty fruit bunches (OPEFBs) are known as one of the most abundant biomasses from palm oil plantations. It is considered as a lignocellulosic residue of palm oil plants, which is regarded as an important agricultural source in Southeast Asian countries. For instance, Malaysia has produced about 15.8 million tonnes of OPEFB annually due to the vigorous activity of oil palm cultivation [10,23]. Upon oil extraction from fresh fruit bunches (FFB), empty fruit bunch (EFB) is attained as biomass with advantageous properties such as being inexhaustible, renewable, biodegradable and environmental friendly [10]. EFB consists of cellulose content ranging from 42.7–65.0%, where as much as 60.6% of this cellulose can be turned into value added products [24]. Cellulose which is derived from EFB can be employed for several applications such as making bioplastic film, paper and polymer composite products. Chemical pre-treatments are usually implemented for cellulose extraction and also to enhance or improve the cellulose dissolution [10,25]. Cellulose pre-treatments are usually carried out on natural fibers to remove the lignin and partly eliminate the hemicelluloses. It also disrupts or breaks the crystalline structure of cellulose, allowing the cellulose to swell and dissolve in suitable solvent [10,25]. Some examples of pre-treatment methods include alkaline treatment [10,25], acid hydrolysis [26], hot compressed water [27] and enzymes [28]. Since cellulosic fibers are innately hydrophilic, they can easily degrade and absorb moisture from the environment. Therefore, chemical modification is applied and utilized to increase hydrophobicity and moisture resistance. Examples of chemical modification methods are silane functionalization [29], acetylation [30] and polymer grafting [31]. However, in the production of regenerated cellulose biocomposite, chemical treatment can also facilitate the regeneration process by reducing the hydrogen bonds of the cellulose and improving its solubility in the ionic liquid [25]. 

To-date, the mechanical and thermal analyses data of the RC film derived from the EFB based cellulose and produced through the use of 1-butyl-3-methylimidazolium chloride (BMIMCl) as ionic liquid and methyl methacrylate (MMA) as chemical treatment not available in the literature, but is truly important to contribute to the progress of the RC biocomposite films intended for use in biodegradable packaging applications. This is because the combination of both chemicals is practical and efficient to produce RC biocomposite with improved performance. BMIMCl has excellent cellulose-dissolving ability while MMA is an industrially relevant chemical with good capability to treat cellulose and improve its dissolution process during the regeneration process. The MMA was previously used to treat chitosan and *Nypa frutican* based-cellulose for the production of biocomposite [32,33]. However, this is the first attempt to utilize MMA in the chemical treatment of the EFB. In order to produce the EFB RC biocomposite film with improved mechanical and thermal properties, the role of MMA as the EFB’s modifier must be clarified while the best EFB loading needs to be determined. This research was aimed at conducting an investigation into all of these aspects, thus revealing the never reported findings for future development of biodegradable plastics.

## 2. Experimental 

### 2.1. Materials

Empty fruit bunch (EFB) fibers produced as biomass from palm oil trees were collected and received from the Malaysian Palm Oil Board (MPOB) in Bangi, Selangor, Malaysia. The EFB fibers were ground using a ring mill into powder form. The average particle size of EFB was 45 microns which was determined using Malvern (Malvern, United Kingdom) particle size analyzer. Microcrystalline cellulose (MCC) was purchased from Aldrich (St. Louise, MO, USA). Sulfuric acid, ethanol and sodium hydroxide (NaOH) were acquired from HmbG Chemicals (Hamburg, Germany). Acetic acid was purchased from BASF Chemical Company (Ludwigshafen, Germany) while sodium chlorite (NaClO_2_) was supplied by Sigma Aldrich (St. Louise, MO, USA). Furthermore, 1-butyl-3-methyl-imidazolium chloride and methyl methacrylate (MMA) were purchased from Merck (Darmstadt, Germany).

### 2.2. Pre-Treatment of EFB

Firstly, EFB powder was treated using 4 wt% of NaOH solution for 1 h at 70 °C. The treatment was performed under mechanical stirring at the speed of 750 rpm. This procedure was repeated 3 times, and the EFB was washed with distilled water and filtered several times after each treatment to remove the soluble alkaline. Then, the alkaline treated EFB was subjected to a bleaching process for further delignification. The bleaching process was carried out in solution containing acetate buffer (solution of 2.7 g NaOH and 7.5 mL glacial acid in 100 mL distilled water), 1.7% w/v aqueous sodium chlorite and distilled water. The EFB was mixed together with the bleaching solution which was stirred for 60 min at the temperature of 70 °C. The bleached fibers were then washed with distilled water and filtered. Acid hydrolysis was carried out using 65 wt% of H_2_SO_4_ at the temperature of 50 °C under mechanical stirring for 45 min. The EFB attained was washed with cold distilled water to stop the acid hydrolysis reaction. The EFB was washed and filtered repeatedly until pH 7 was reached. The filtered EFB was dried in an oven for a day at 70 °C.

### 2.3. EFB Modification

Chemical treatment was carried out on pre-treated EFB using methyl methacrylate (MMA). Firstly, the MMA solution was prepared by dissolving 3% of MMA in ethanol (v/v). Then, the EFB was slowly added into the MMA solution and the mixture was stirred continuously for 1 h. The solution was left overnight. The next day, the EFB was filtered and dried in an oven at 70 °C for 24 h.

### 2.4. Preparation of EFB Regenerated Cellulose (RC) Biocomposite Films

The concept of this RC biocomposite is the same as self-reinforced composite (SRC). Both EFB cellulose and MCC were used as the reinforcement and matrix when they were partially dissolved in the 1-butyl-3-methylimidazolium chloride (BMIMCl) solvent system. The dissolved cellulose was transformed into the matrix phase surrounding the remaining non-dissolved cellulose. EFB RC biocomposite films with 1, 2, 3 and 4 wt% EFB (untreated and MMA treated), plus a constant amount of microcrystalline cellulose (MCC) (3 wt%) were prepared for this study. BMIMCl was used as solvent in the regeneration process of the EFB cellulose. Firstly, the BMIMCl was heated in order to dissolve it into liquid form. Furthermore, 3 wt% of MCC and 1 wt% of EFB was dispersed in BMIMCl. The mixture was heated at the temperature of 100 °C and stirred for 30 min until a homogeneous cellulose solution was achieved. The cellulose solution was cast onto a glass plate mold. As the film sample developed, it was soaked in a water bath to allow the diffusion of ionic liquid out of the sample. Water was changed a few times to ensure complete removal of ionic liquid from the film sample. The film attained was dried in an oven for 24 h at 50 °C. Similar steps were repeated to produce the EFB RC biocomposite films with different contents of untreated and treated EFB. Note that the purpose of adding MCC into the mixture was to provide a strengthening effect to the RC biocomposite films. Preliminary work was done in which the RC films were produced without the addition of MCC. Unfortunately, the films produced were too brittle. In addition, we have tried to prepare the film with 0% EFB; however, no good film has been successfully produced. These factors were the reason for preparing the RC biocomposite films with both MCC and EFB. As MCC was added in constant amounts (3 wt%) in all the RC biocomposite films, the changes in the morphology and properties of the films reported in the discussion part were assumed due to the EFB contents.

**Figure 1:**
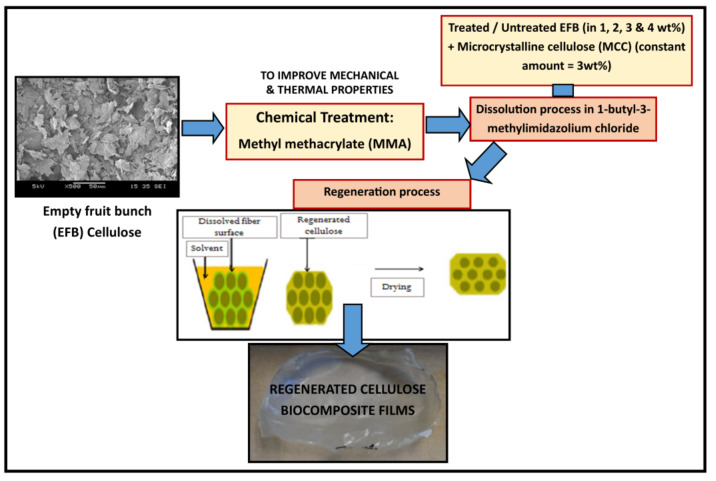
Procedures to prepare EFB RC biocomposite films.

The overall process to produce the EFB RC biocomposite films is illustrated in the schematic diagram in Figure 1.

## 3. Texting and Characterization

### 3.1. Tensile Testing

Instron Universal Testing Machine (Norwood, MA, USA), model 5590 was used to carry out the tensile test which was based on ASTM D 882. The tensile test was operated at the speed of 10 mm/min. The EFB RC biocomposite films were cut into rectangular shapes with dimensions of 15 mm × 100 mm. The thickness of the sample was measured using a digital vernier caliper along its gauge length area three times (at center, upper and bottom sides). The mean value was taken and recorded. Five replicates of each material were used for the tensile test, and the average values of tensile strength, modulus of elasticity and elongation at break were attained and recorded. Statistical analysis was performed using two-tailed Student’s t-test for unpaired data to compare among materials. A significance level of 0.05 or less was accepted as statistically significant.

### 3.2. Scanning Electron Microscopy (SEM)

Scanning electron microscope (SEM) model JEOL JSM-6460LA (Akishima, Japan) was utilized to analyze and study the morphology of EFB RC biocomposite films. The fractured surface of the biocomposite film was subjected to morphological analysis to study the deformation behavior of the film samples. The films were coated with a thin layer of palladium for conductive purpose. Scanning was done at a voltage of 10 kV.

### 3.3. X-Ray Diffraction (XRD)

XRD analysis was conducted using a Bruker DS X-ray diffractometer (Billerica, MA, USA) utilizing X-ray sources of Cu-Kα radiation (λ = 1.5418 Å). The analysis was carried out under normal atmospheric conditions and at ambient temperature. Samples used for XRD testing were strips of films with dimensions of 10 mm × 15 mm [32]. The crystallinity index (CrI) for both untreated and treated EFB RC biocomposite films were calculated based on [10,34]:

Crystallinity Index (CrI) = (I/I’) / (I) × 100(1)

where I = the height of peak assigned to (200) planes, measured in the range 2θ = 20–23°, and I’ = the height of peak assigned to (100) planes, located at 2θ = 12–16°.

### 3.4. Fourier Transform Infrared (FTIR)

A Perkin Elmer L1280044 FTIR spectrometer (Waltham, MA, USA) was used to study the functional groups of untreated and treated EFB RC biocomposite films. Four scans were conducted on the films in the wavenumber range from 4000–650 cm^–1^ with resolution of 4 cm^–1^ using attenuated total reflectance (ATR) method.

### 3.5. Thermogravimetric Analysis (TGA)

TGA Pyris Diamond Perkin Elmer (Waltham, MA, USA) was used to analyze the thermal behavior of both untreated and treated EFB RC biocomposite films. The film samples used were weighed to about 5 ± 2 mg. The films were placed in aluminum pans and heated from 25 to 700 °C with a heating rate of 10 °C/min. The testing was performed in nitrogen atmosphere with nitrogen flow rate of 50 mL/min.

## 4. Results and Discussion

### 4.1. Tensile Properties

**Figure 2:**
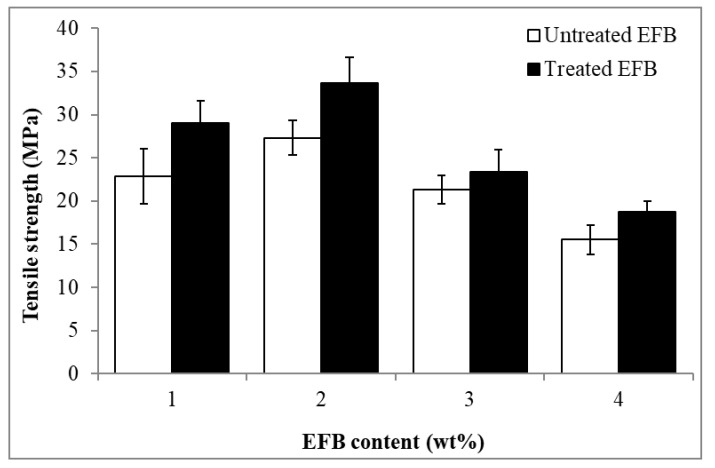
Effect of EFB content on tensile strength of untreated and MMA-treated EFB RC biocomposite films.

Figure 2 shows the effect of EFB content on the tensile strength of the untreated and MMA treated EFB-based RC biocomposite films (EFB RC biocomposite). The tensile strength for both untreated and treated EFB RC biocomposite films demonstrated similar trends in which the value increased initially when the EFB content increased from 1 to 2 wt%. Further increase in the EFB content resulted in the decrease of the tensile strength of the EFB RC. Large accumulations of EFB particles led to non-homogeneous dispersion of the EFB during the formation of the EFB RC biocomposite film. This would develop more stress concentrated areas that hasten the failure of the biocomposite’s tensile deformation. The highest tensile strength value (34 MPa) was obtained when 2 wt% MMA treated EFB was employed to form the RC biocomposite. Obviously, at similar EFB content, the treated EFB RC biocomposite films obtained higher tensile strength compared to the untreated biocomposite films. For instance, when 2 wt% MMA treated EFB was used to form the RC biocomposite; a 26% improvement in tensile strength was obtained vs. the untreated RC biocomposite film. It is proven that the treatment of EFB with MMA has enhanced and improved the interfacial adhesion between the non-dissolved (filler phase) and dissolved (matrix phase) cellulose, allowing greater stress transferring mechanisms throughout the biocomposite structure.

**Figure 3:**
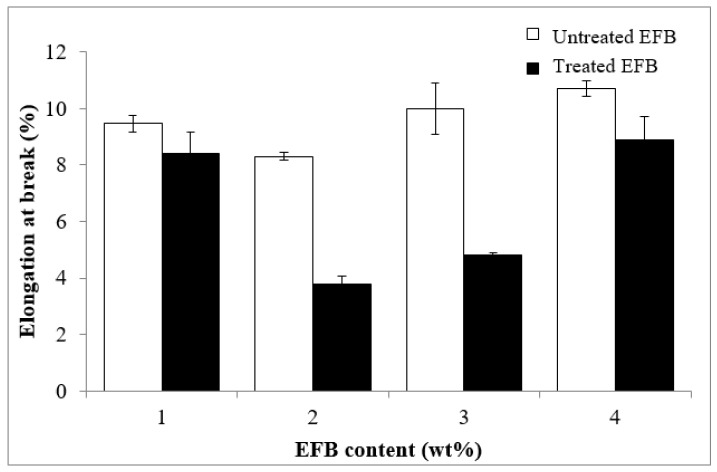
Effect of EFB content on elongation at break of untreated and MMA-treated EFB RC biocomposite films.

Figure 3 displays the effect of EFB content on the elongation at break of the untreated and MMA treated EFB RC biocomposite films. Generally, the elongation at break values of the biocomposites does not show any clear trend. The fluctuating of the elongation at break value could be related to environmental influences such as moisture, absorption rate of the film and different levels of void content due to random dispersion of the EFB particles in the biocomposite structure. These factors affect the flexibility and conformation of the biopolymer chains when subjected to tensile deformation. In addition to that, the use of single material (EFB cellulose which functioned as both matrix and reinforcement) to form the biocomposite can cause an uncontrollable portion of the dissolved cellulose (matrix) and undissolved cellulose (filler) in the RC biocomposite structure. In this case, the “real composition” of filler phase may not be proportional with the EFB content, thus the portion that contributes to elasticity may also vary. These reasons might explain the unexpected elongation at break results of these biocomposite films. However, regardless of the EFB content, the MMA treated films exhibit lower elongation at break compared to the untreated biocomposite films. Enhancement in the interfacial bonding between matrix and filler obtained through the chemical treatment has restrained the flexibility and mobility of the polymer chain.

**Figure 4:**
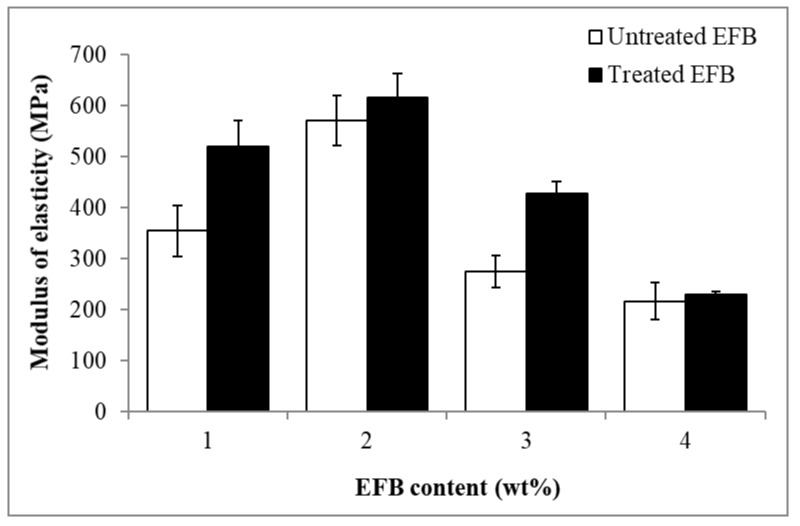
Effect of EFB content on modulus of elasticity of untreated and MMA-treated EFB RC biocomposite films.

The effect of EFB content on the modulus of elasticity of the untreated and MMA treated EFB RC biocomposite films is illustrated in Figure 4. The modulus of elasticity for the untreated and treated EFB RC biocomposite films increased as the EFB content increased from 1 to 2 wt%. Addition of more EFB content (3 and 4 wt%) resulted in the declination of modulus of elasticity for both types of film. At 1, 2 and 3 wt% EFB content, the treated RC films demonstrated higher modulus of elasticity than the untreated biocomposite films. As mentioned earlier, MMA treatment of EFB has improved the matrix-filler interactions, thereby increasing the stiffness of the biocomposite films. The higher stiffness required higher stress to prompt movement, thereby resulting in the increase in the modulus of elasticity of the biocomposite films. However, when 4 wt% EFB was employed, the modulus of elasticity value of the RC biocomposite film with the untreated EFB statistically shows no significant difference to the RC biocomposite film with the MMA-treated EFB. Even though the EFB has been treated with the MMA, the high content and overcrowding of the cellulose might hinder the aligning of the cellulosic molecular chains in an orderly manner during the regeneration process, hence more flexible chains are formed. This results in no significant increase in the modulus of elasticity of the treated EFB RC biocomposite when compared with the untreated one.

### 4.2. X-Ray Diffraction (XRD)

**Figure 5:**
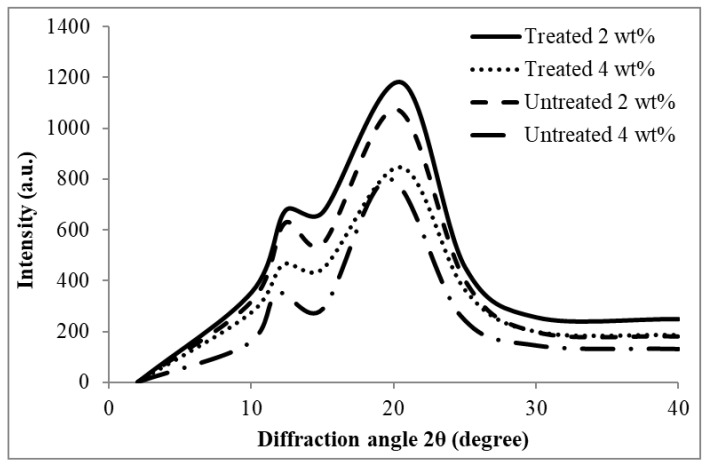
XRD pattern of untreated and MMA-treated RC biocomposite films containing 2 and 4 wt% EFB.

Figure 5 shows the XRD pattern of the untreated and treated RC biocomposite films with MMA at 2 and 4 wt% of EFB content, while Table 1 summarizes their crystallinity index (CrI). The diffraction peaks of all the samples were observed in the 2θ range of 11.0–13.0° and 19.0–21.0°. These peaks suggest the cellulose II structure, as stated by Zhang et al. [35]. The intensity of the peaks of the treated films is higher compared to the untreated biocomposite films, thus the CrI is also higher. This was due to the treatment of EFB with MMA which has improved and enhanced the dissolution and regeneration of cellulose, thus a higher degree of cellulose II was formed.

### 4.3. Morphology Study

**Figure 6:**
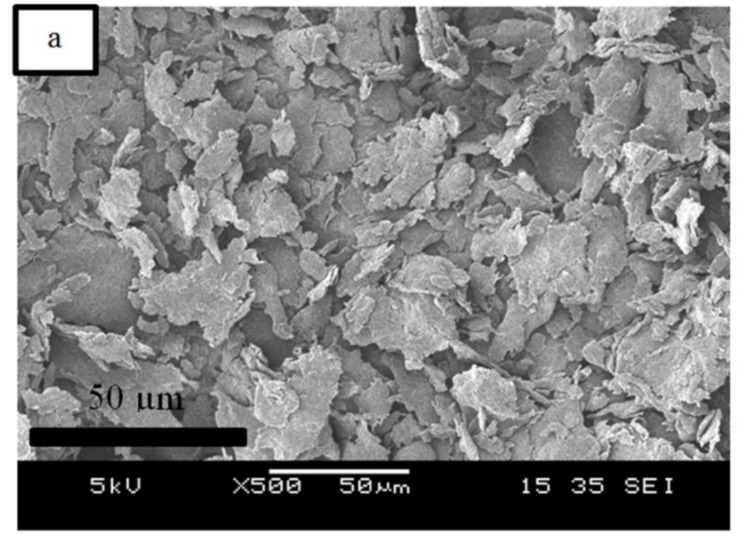

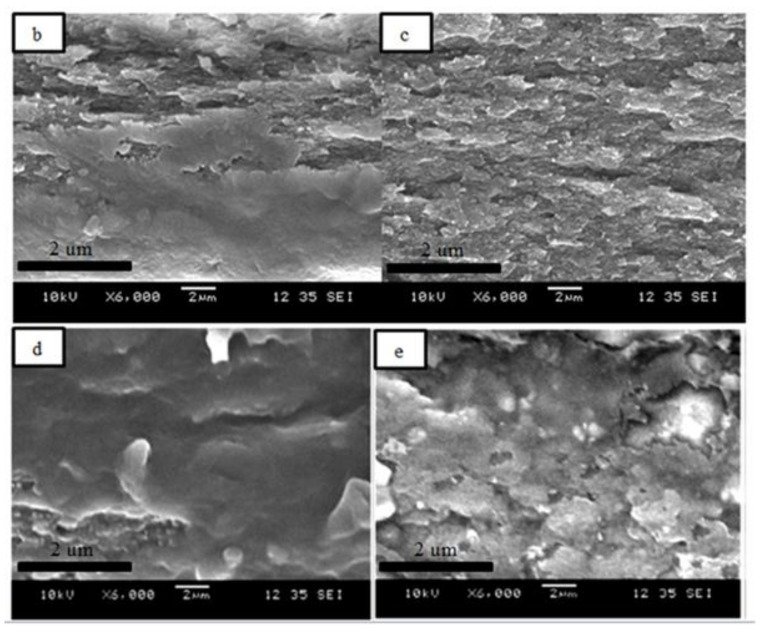
(**a**) SEM of EFB powder. Tensile fractured surface of (**b**) untreated RC biocomposite film containing 2 wt% EFB, (**c**) untreated RC biocomposite film containing 4 wt% EFB, (**d**) treated RC biocomposite film containing 2 wt% EFB and (**e**) treated RC biocomposite film containing 4 wt% EFB as observed through SEM.

Figure 6a, and Figure 6b–e represent the SEM images of the EFB powder and fractured surface of the RC biocomposite films at 2 and 4 wt% of EFB content, respectively. The SEM image of the EFB powder indicates that it contains particles with irregular shape. Upon regeneration process, the particulate form of the EFB disappeared as observed through the images of the biocomposite films (Figure 6b–e). A smoother and more homogeneous surface can be observed in the EFB RC biocomposite films that contain 2 wt% EFB as compared to the ones containing 4 wt% EFB (untreated and MMA treated). This shows that, lower content of EFB (2 wt%) resulted in better dissolution and dispersion of the EFB in the structure of the RC biocomposite. The presence of higher EFB content (4 wt%) resulted in poorer dispersion of the non-dissolved EFB (filler phase) and lower rate of dissolution of the regenerated cellulose (matrix phase). Similar results were attained by Liu et al. [12], which concluded that the homogeneous surface was obtained in films regenerated from cellulose solution containing low cellulose content. Furthermore, it can also be witnessed that the chemical treatment of the EFB using MMA resulted in a more homogeneous and smoother surface morphology of the RC biocomposite. The micrographs also indicate that fewer aggregates were observed. This implied that the reinforcement was well-surrounded by the regenerated cellulose matrix, due to improved interactions between both filler and matrix phases. The untreated RC biocomposite film with 4 wt% of EFB content (Figure 6c) illustrates a rough and heterogeneous morphology, which could be the cause of its tensile property reduction as mentioned previously.

### 4.4. Thermal Gravimetric Analysis

**Figure 7:**
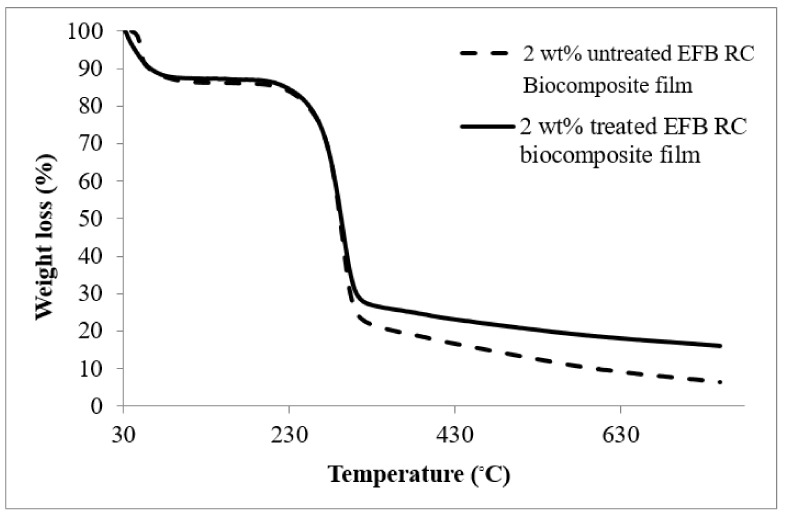
Thermal gravimetric analysis (TGA) curves of untreated and MMA-treated EFB RC biocomposite films containing 2 wt% EFB.

Thermal gravimetric analysis (TGA) curves of the untreated and MMA-treated EFB RC biocomposite film containing 2 wt% of EFB were illustrated in Figure 7, while Table 2 tabulated the TGA data for all those materials. At the early stages of the thermal decomposition process (which is below 100 °C), the weight loss was due to the volatilization and vaporization of water [34,35]. At temperature range of 95–191 °C, the weight loss for both types of biocomposite film remained almost unchanged. This could be due to the removal of the remaining hemicellulose and extractives upon the alkaline treatment [34]. At temperatures higher than 190 °C, the decomposition of the RC biocomposite films generally occurred in two stages and this trend was also observed by Yeng et al. [34] and Reddy et al. [35]. At the first stage, a sharp weight loss can be observed in the temperature range of 191–282 °C for the treated RC film and 182–285 °C for the untreated film. This is associated with the degradation process of the crystalline cellulose. The second degradation stage occurred at 289–427 °C for treated film and 288–425 °C for untreated film, indicating that the cellulose almost completely burnt due to the continuing thermal oxidative degradation [35,36]. Apparently, the treated biocomposite films exhibit higher maximum degradation temperature (T_dmax_) as opposed to the untreated films. This is ascribed to the better compatibility between the matrix and filler phase. Most obvious at temperatures between 300 and 600 °C, the treated films demonstrate lower weight loss. This suggests that the MMA treatment of the EFB can improve the thermal stability of the EFB RC biocomposite film. Other researchers also realized the benefit of chemical treatment of fiber in improving its thermal stability. For instance, Chen et al. [37] have analyzed and reported the thermal properties of the cellulose fibers from rice straw, and concluded that the treated cellulose fibers possess higher degradation temperatures and higher thermal stability than the untreated ones.

### 4.5. Fourier Transform Infrared Spectroscopy Analysis (FTIR)

**Figure 8:**
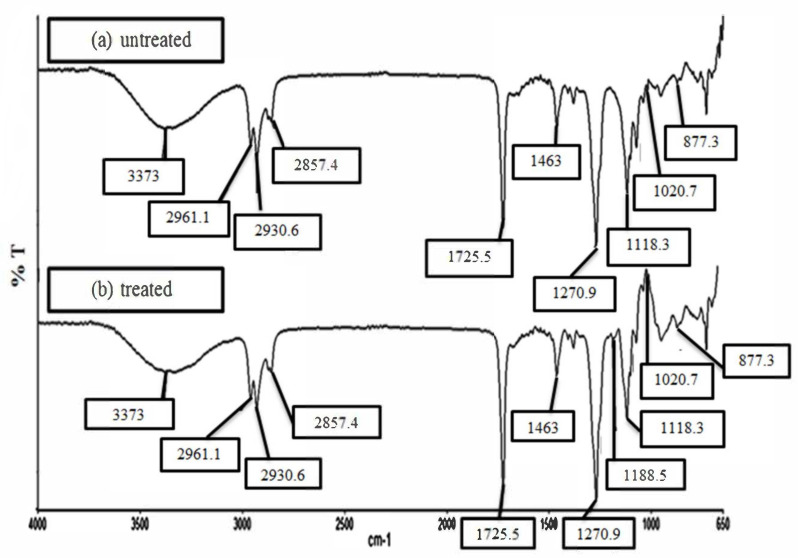
FTIR spectra of (**a**) untreated and (**b**) treated RC EFB biocomposite films with MMA.

**Figure 9:**
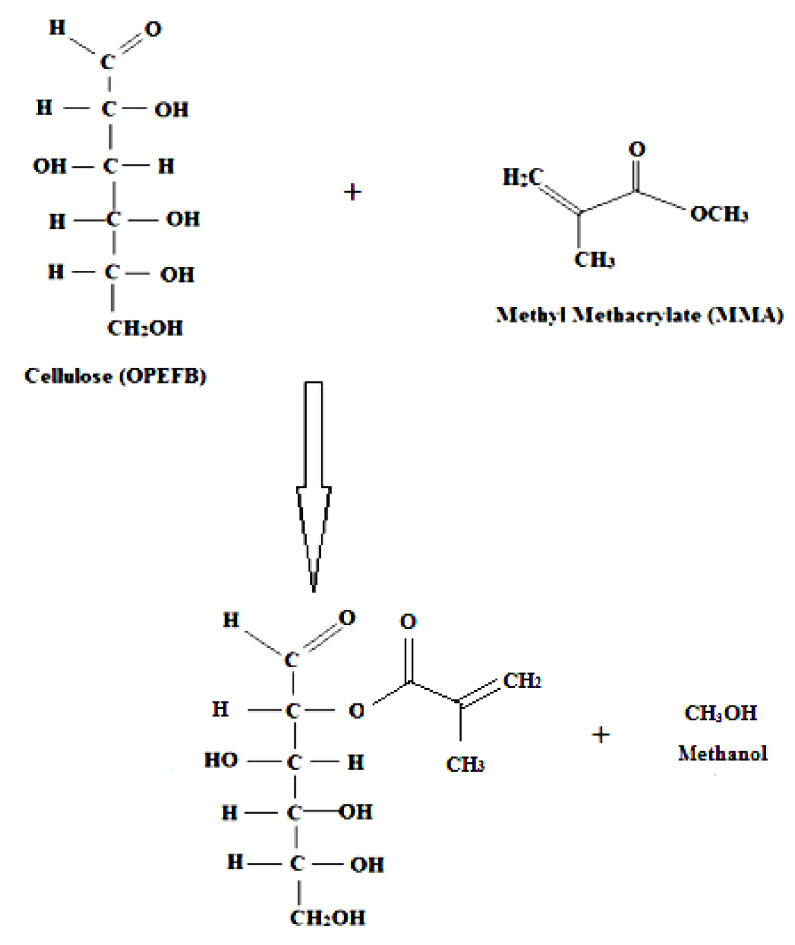
The schematic reaction between EFB and MMA.

Figure 8a,b illustrates the FTIR spectra of the untreated and MMA-treated EFB RC biocomposite films. Both untreated and treated EFB RC biocomposite films demonstrate similar peaks, suggesting that both films have comparable chemical compositions. The broad peak at 3373 cm^–1^ relates to the appearance of –OH stretching vibration. The band which can be seen at 2961 and 2930 cm^–1^, is attributed to the C-H group’s stretching vibration [10,38]. Vibration of CH_2_ groups is implied by the peak at 2857 cm^–1^. The peak at 1725 cm^–1^ corresponds to the carbonyl groups (C=O) appears due to the chemical treatment of the EFB using MMA. The reason for the appearance of this peak in the spectra of both untreated and treated EFB RC biocomposite films is that there might be some residual of the lignin which also exhibits these functional groups. The absorption band observed at 1463 cm^–1^ relates to the CH_3_ and CH_2_ deformation vibration, while the peak at 1270 cm^–1^ displays the presence of C-O bond stretching vibration [10,38]. The absorption band detected at 1188 cm^–1^ for the treated biocomposite films indicated the anti-symmetric C-O-C stretch from the ester [10]. The band which is seen at 1118 cm^–1^ suggests the C-O-C pyranose ring skeletal vibration. The appearance of the band at 1020 cm^–1^ in both untreated and treated films indicates the presence of C-O bond stretching vibration of C-O-C linkage in the anhydroglucose unit (AGU). The band at ~877 cm^–1^ is associated with β-glycosidic linkages between glucose in the EFB cellulose as a result of ring vibration and –OH bending of the glycosidic C-H group. This type of response can only be seen in the regenerated cellulose. Figure 9 demonstrates the schematic reaction between EFB and MMA. Upon chemical treatment, the hydrogen bonds of the EFB cellulose reduced due to replacement of its –OH group with the ester group of the MMA. As a result, the EFB cellulose can be better dissolved in the solvent, leading to an improved regeneration process and greater interphase bonding between the filler phase and matrix phase of the EFB RC biocomposite. As observed through the SEM analysis, more homogeneous RC biocomposite was obtained that also led to the improved mechanical and thermal properties of the EFB RC biocomposite film.

## 5. Conclusions

The effects of EFB content and chemical treatment of EFB using methyl methacrylate (MMA) on the mechanical and thermal properties of the EFB RC biocomposite films were studied. Results demonstrated that the treated EFB films exhibit higher and greater tensile strength and modulus of elasticity, but lower elongation at break compared to the untreated films. The treated EFB RC films also attained higher crystallinity index. Surface morphology of the treated biocomposite films indicates smoother surface due to the MMA treatment. Thermal analysis demonstrates that the treated biocomposite film possesses higher T_dmax_ and lower weight loss at 600 °C. This suggests that the treated biocomposite film has greater thermal stability. The presence of ester in the treated EFB RC biocomposite films with MMA was proven and affirmed by FTIR analysis. The substitution of the –OH unit of the EFB cellulose with the ester unit of the MMA has facilitated the dissolution process of the EFB during the regeneration process due to weakening of the hydrogen bonding of the cellulose. A more homogeneous biocomposite structure developed which led to the improvement of both tensile properties and thermal stability of the EFC RC biocomposite film.

## Figures and Tables

**Table 1:** The crystallinity index (CrI) of untreated and MMA-treated EFB RC biocomposite films containing 2 and 4 wt% EFB.

Biocomposite Films	Untreated	Treated
	CrI (%)	CrI (%)
2 wt% EFB RC	41.9	45.0
4 wt% EFB RC	38.5	40.7

**Table 2:** TGA data of EFB RC biocomposite films containing 2 wt% EFB.

Biocomposite Films	T_dmax_ (°C)	Weight Loss (%)	
		300 °C	600 °C
2 wt% untreated EFB RC	293	65.1	81.6
2 wt% treated EFB RC	295	59.3	78.6
